# Nearshore marine biodiversity of Osa Peninsula, Costa Rica: Where the ocean meets the rainforest

**DOI:** 10.1371/journal.pone.0271731

**Published:** 2022-07-28

**Authors:** Alan M. Friedlander, Enric Ballesteros, Odalisca Breedy, Beatriz Naranjo-Elizondo, Noelia Hernández, Pelayo Salinas-de-León, Enric Sala, Jorge Cortés

**Affiliations:** 1 Pristine Seas, National Geographic Society, Washington, DC, United States of America; 2 Hawaiʿi Institute of Marine Biology, University of Hawaiʿi, Kāneʻohe, Hawaiʿi, United States of America; 3 Centre d’Estudis Avancats de Blanes-CSIC, Blanes, Girona, Spain; 4 Centro de Investigación en Ciencias del Mar y Limnología, Universidad de Costa Rica, San José, Costa Rica; 5 Asociación Conservación Osa, Puerto Jiménez, Golfito, Costa Rica; 6 Charles Darwin Research Station, Charles Darwin Foundation, Puerto Ayora, Galápagos Islands, Ecuador; Universita degli Studi di Genova, ITALY

## Abstract

Osa Peninsula in remote southwest Costa Rica harbors 2.5% of global terrestrial biodiversity in only 1,200 km^2^ and has the largest remaining tract of Pacific lowland wet forest in Mesoamerica. However, little is known about the marine ecosystems of this diverse region. Much of the coastline consists of soft sediment exposed to strong wave action. Three major hard bottom habitat types define this region, including: 1) coral reefs around Isla del Caño Biological Reserve, a no-take marine protected area (MPA) of 52 km^2^, 2) coastal rocky reefs and islets along the peninsula, including Corcovado National Park, and 3) submerged pinnacles just outside the Isla del Caño MPA. Average coral cover at Isla del Caño was 21%, composed primarily of *Porites lobata* and *Pocillopora elegans*. In contrast, coastal rocky reefs were dominated by turf algae (39.8%) and macroalgae (20.7%) with low coral cover (1.1%). Submerged pinnacles were dominated by crustose coralline algae (33.3%) and erect coralline algae (25.7%). Fish assemblage characteristics (species richness, abundance, biomass) were significantly higher at the pinnacles compared to the other habitats and was dominated by schooling species such as *Haemulon steindachneri*, and the herbivores *Kyphosus ocyurus*, and *Acanthurus xanthopterus*. Top predators, primarily *Triaenodon obesus*, *Caranx sexfasciatus*, and *Lutjanus argentimaculatus*, were also most abundant at these pinnacles and accounted for the largest differences in fish trophic structure among habitats. Despite Isla del Caño being fully protected from fishing, biomass was similar to fished areas along the coast and lower than the adjacent submerged pinnacles outside the reserve. Similarly, Corcovado National Park includes 20.3 km^2^ of no-take MPAs; however, there is limited enforcement, and we noted several instances of fishing within the park. The unique configuration of healthy offshore coral reefs and pinnacles connected to coastal habitats provides corridors for many species including large predators such as sharks and other marine megafauna, which warrants additional protection.

## Introduction

Osa Peninsula in the remote southwest corner of Costa Rica is recognized as one of the most biodiverse places on earth relative to its size (~ 1,200 km^2^), with 2.5% of the world’s known terrestrial species of plants and animals [1]. Once an island in the Pacific, the peninsula evolved in isolation until it merged with mainland Costa Rica two million years ago [2]. One of the last places in Costa Rica to be settled by large human groups, the Osa Peninsula has the largest remaining tract of Pacific lowland wet forest in Mesoamerica [1], which extends all the way to the Pacific Ocean. High species richness has also been reported from Osa’s marine-coastal ecosystems [3]. Within this area are the largest mangrove areas in the country, protected by the Térraba-Sierpe National Wetland [4], and the only tropical fjord with anoxic waters, Golfo Dulce, which separates Osa Peninsula from the Costa Rican mainland [5]. This embayment of tectonic origin, despite being anoxic at depths > 60 m, has a high diversity of marine fauna [6–8], and is important for marine mammals, sharks, and seabirds [3, 9, 10].

In addition, there are a diversity of marine ecosystems from shallow to deep waters, including rocky outcrops scattered along the coast, and submerged pinnacles and various types of hard and soft bottom habitats in a wide range of depths, making this region one of the most diverse in the country [11]. The Osa Peninsula is an important breeding habitat for highly migratory species such as scalloped hammerhead sharks (*Sphyrna lewini*) [12] and humpback whales (*Megaptera novaeangliae*) [3]. However, the biodiversity and marine-coastal environments have been degraded by various human activities, mainly due to the rapid expansion of agricultural and tourism development, land contamination, and overexploitation of natural resources [13].

Covering much of the western portion of the Osa Peninsula is the Corcovado National Park (422.3 km^2^ including the marine portion). Corcovado is an icon of terrestrial conservation since its creation in 1975, consisting of lowland rainforest, highland cloud forest, jolillo palm forest, mangrove swamps, marine coasts, and beach habitats [14]. The marine boundaries of this park extend ~ 40 km along the coast and out to 500 m (covering 20.4 km^2^), which were added in 1980 mainly to protect sea turtles nesting sites although fishing is also prohibited [15]. Several sections along the shallow coast have well developed coral reefs; however, much of nearshore ecosystem has been poorly explored [16, 17].

The Isla del Caño Biological Reserve, located ~ 15 km off the coast, is a no-take marine protected area (MPA, 52 km^2^), which is home to the most extensive coral reefs on the Pacific coast of Costa Rica [18–21]. The corals of Isla del Caño were severely impacted by the 1982–1983 El Niño event, resulting in losses of up to 50% of live coral cover [18, 19, 22]; however, subsequent El Niño events in the 1990s impacted these reefs to a lesser extent [23]. Isla del Caño has a high richness of octocorals with at least 12 species known from 10 to 30 m. Between 30 and 50 m, octocoral species richness declines; however, there is a unique faunal assemblage in these deeper depths and a new species to science was recently described that suggests a higher number of octocoral species are still to be discovered [24].

According to the marine conservation gap analysis conducted by the Costa Rican National System of Conservation Areas (SINAC), the Osa region is one of the main areas of the country where significant gaps were found [11]. To date, these conservation gaps have not been effectively addressed, and marine conservation efforts occur in only two small MPAs. Because of the high terrestrial biodiversity of Osa Peninsula, it is important to also understand the adjacent marine environment and its importance in land-sea linkages. The marine corridor adjacent to Osa Peninsula is largely unknown and the major goals of this study were to describe the marine habitats and ecosystems of the Osa region, identify key ecosystem elements, and help inform proposals for increased marine protection for the region.

## Materials and methods

### Ethics statement

Data were collected by all authors in a collaborative effort. Non-invasive research was conducted, which included photographs, video, and visual estimates described in the methods below. The Costa Rica National System of Conservation Areas (SINAC), Osa Conservation Area office (ACOSA), and Costa Rican Fisheries and Aquaculture Institute (INCOPESCA) granted all necessary permissions to conduct this research. No vertebrate sampling was conducted and therefore no approval was required by any Animal Care and Use Committee. Our data are publicly available at Data Dryad: https://doi.org/10.5061/dryad.np5hqbzws

### Habitats and sampling locations

**Fig 1:**
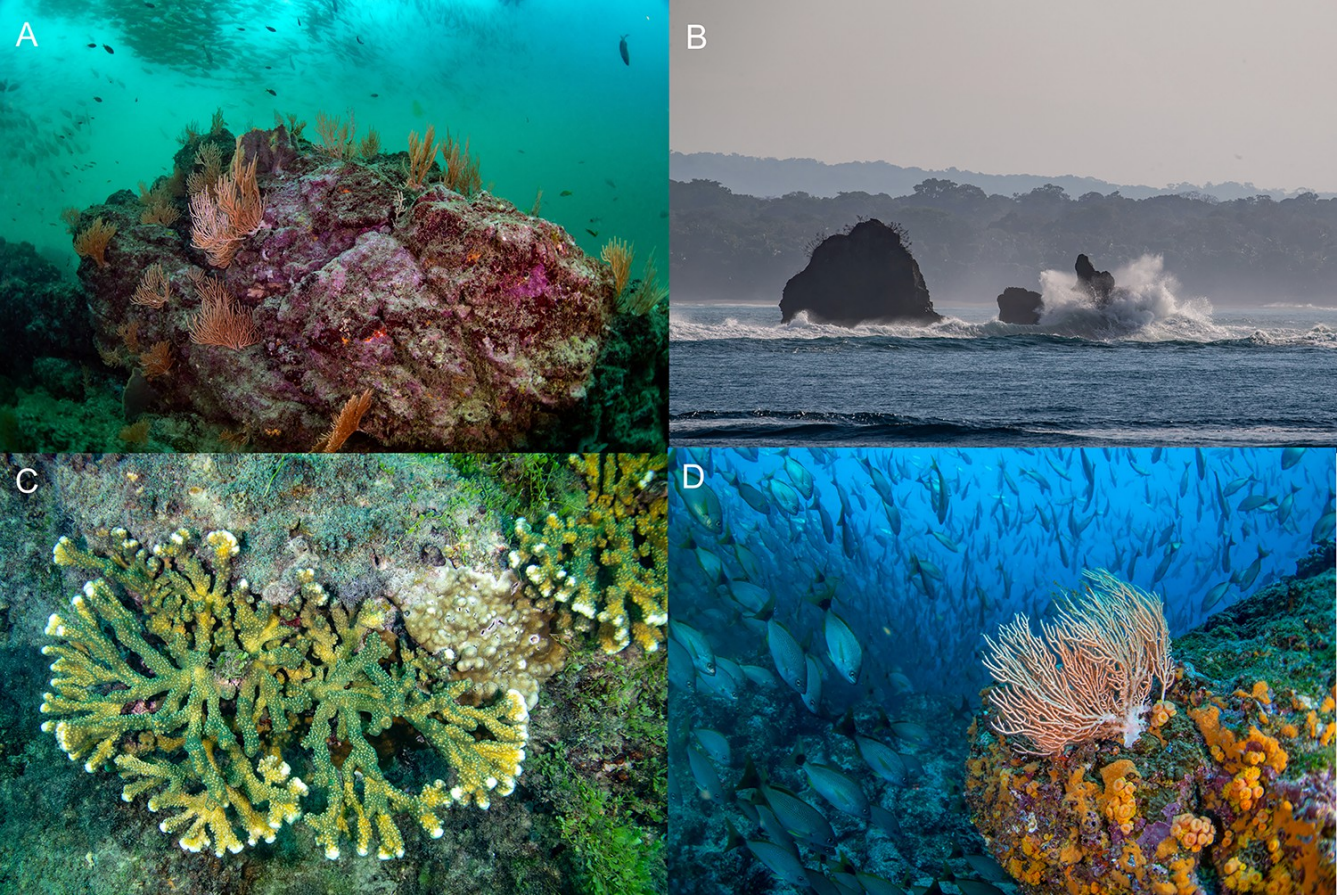
Major habitat types observed around the Osa Peninsula. A. Coastal rocky reef, B. Roca Corcovado, a coastal islet, C. Coral reefs around Isla del Caño Biological Reserve, and D. Bajo Diablo, a submerged pinnacle outside the Isla del Caño Biological Reserve. All photos courtesy of Manu San Félix.

Precipitation in the region averages 4,000 to 6,000 mm y^−1^ with distinct rainy (May–December) and dry seasons (January–April) [25]. Sampling was conducted from 11 to 22 March 2019, during the dry season. Habitat strata were defined *a priori* based on previous knowledge of the area [16, 17, 21] and included: 1) coral reefs around Isla del Caño Biological Reserve (IC), 2) coastal rocky reefs and islets along the Osa Peninsula, including Corcovado National Park (COAST), and 3) submerged pinnacles just outside the Isla del Caño MPA (PIN) (Fig 1). Much of the coastline along the Osa Peninsula consists of soft sediment exposed to strong wave action and large river inputs [26], which limited sampling in this region. The coastal rocky reefs and islets that do exist are mostly shallow (< 20 m deep) and dominated by barnacles, crustose coralline algae with few scleractinian corals [16]. In contrast, Isla del Caño, which is located ~ 15 km of the coast, has minimal coastal influences and harbors some of the best developed coral reefs in Costa Rica [19, 21]. The pinnacles just outside the Isla del Caño MPA come to within 10–15 m of the surface and represent a unique habitat within the region.

Sampling was stratified by depth strata (shallow = ~ 10 and deep = ~ 20 m). Efforts were made to sample both strata at each site when possible. However, deep habitat was not always present at some of the COAST sites and shallow sites were not always present at PIN sites, resulting in a slightly unbalanced sampling design.

### Underwater visual censuses

For algae, corals, and other sessile invertebrates we used a line-point intercept methodology along each transect, recording the species or taxa found every 20 cm on the measuring tape. Five 10-m long transects were conducted at each depth strata (~ 10 and 20 m). Mobile invertebrate densities were quantified in 50 x 50 cm quadrats, with 25 quadrats per depth stratum. All individuals were identified to the lowest possible taxa.

Within each depth strata fish transects were conducted along three 25-m long transects laid along isobaths within a homogeneous habitat, with 5 m separating each transect. At each survey site, a scuba diver counted and sized all fishes of total length (TL) ≥20 cm within a 4 m wide strip on an initial ‘‘swim out” while the transect line was laid (transect area = 100 m^2^), and all fishes of TL <20 cm were counted in a 2 m wide strip along the transect line on the way back (transect area = 50 m^2^). Total fish lengths were estimated to the nearest cm. All fishes were identified to the species level based on Robertson and Allen [27] and categorized into four trophic groups: top predators (trophic level ≥ 4.0), herbivores, secondary consumers, and planktivores using FishBase (http://www.FishBase.org). Valid scientific names were verified according to Fricke et al. [28].

The biomass of individual fishes was estimated using the allometric length-weight conversion: W = aTL^b^, where parameters a and b are species-specific constants, TL is total length in cm, and W is weight in grams. Length-weight fitting parameters were obtained from FishBase (www.fishbase.org). The sum of all individual weights and numerical densities was used to estimate biomass density by species. Species diversity was calculated using the Shannon-Wiener diversity index [29]: H’ = Σ (*p*_*i*_ × ln *p*_*i*_), where *p*_*i*_ is the proportion of all individuals counted that were of species *i*.

### Statistical analyses

Sessile benthic taxa were grouped into functional groups for higher level analyses. These included: crustose coralline algae (CCA), scleractinian coral, soft corals (Alcyonacea), erect coralline algae, cyanobacteria, macroalgae, sponges, turf, other invertebrates (e.g., mollusks, ascidians, hydroids). Drivers of sessile benthic communities, mobile invertebrate communities, and fish assemblage structures were investigated using permutation-based multivariate analysis of variance (PERMANOVA). Bray–Curtis similarity matrices were created from percent cover of sessile benthic taxa, mobile invertebrate abundance, and biomass of fish taxa. Prior to analyses, sessile benthic taxa cover was arcsine-sqrt transformed, mobile invertebrate community structure was ln(x+1) transformed, and fish biomass was 4^th^ root transformed. Fixed factors for the PERMANOVA were depth (shallow = 10 m, deep = 20 m) and habitat (IC, PIN, COAST).

Interpretation of PERMANOVA results was aided using individual analysis of similarities (ANOSIM), and similarity of percentages analysis (SIMPER) of species responsible for such patterns [30]. ANOSIM is a permutation-based hypothesis testing analysis of similarities that generates an R statistic that is on a scale from 0 or negative value (identical assemblages) to 1 (completely dissimilar assemblages). The resulting P value indicates the probability that the two assemblages come from a similar distribution [31]. Pairwise ANOSIM R statistics represent comparisons that are well separated (R> 0.75), overlapping but clearly different (R > 0.5), or barely separable at all (R< 0.25). SIMPER was used to determine the sessile benthic taxa, mobile invertebrate taxa, and fish species most responsible for the percentage dissimilarities between habitats using Bray-Curtis similarity analysis of hierarchical agglomerative group average clustering. Principal Coordinate Analysis (PCO) was used to compare sessile benthic, mobiles invertebrate, and fish assemblage structures among habitat types.

The number of sessile benthic taxa and fish assemblage characteristics (e.g., species richness, number of individuals, biomass, diversity) were compared using generalized linear mixed models (GLMMs) with a Poisson distribution and log-link function with habitat, depth strata, and their interaction treated as fixed factors. Site was treated as a random effect in the models. Contrasts between fixed factors were conducted using likelihood ratio tests (α = 0.05). The overdispersion parameter was estimated by Pearson’s χ^2^/df.

## Results

### Nearshore ecosystems

**Fig 2:**
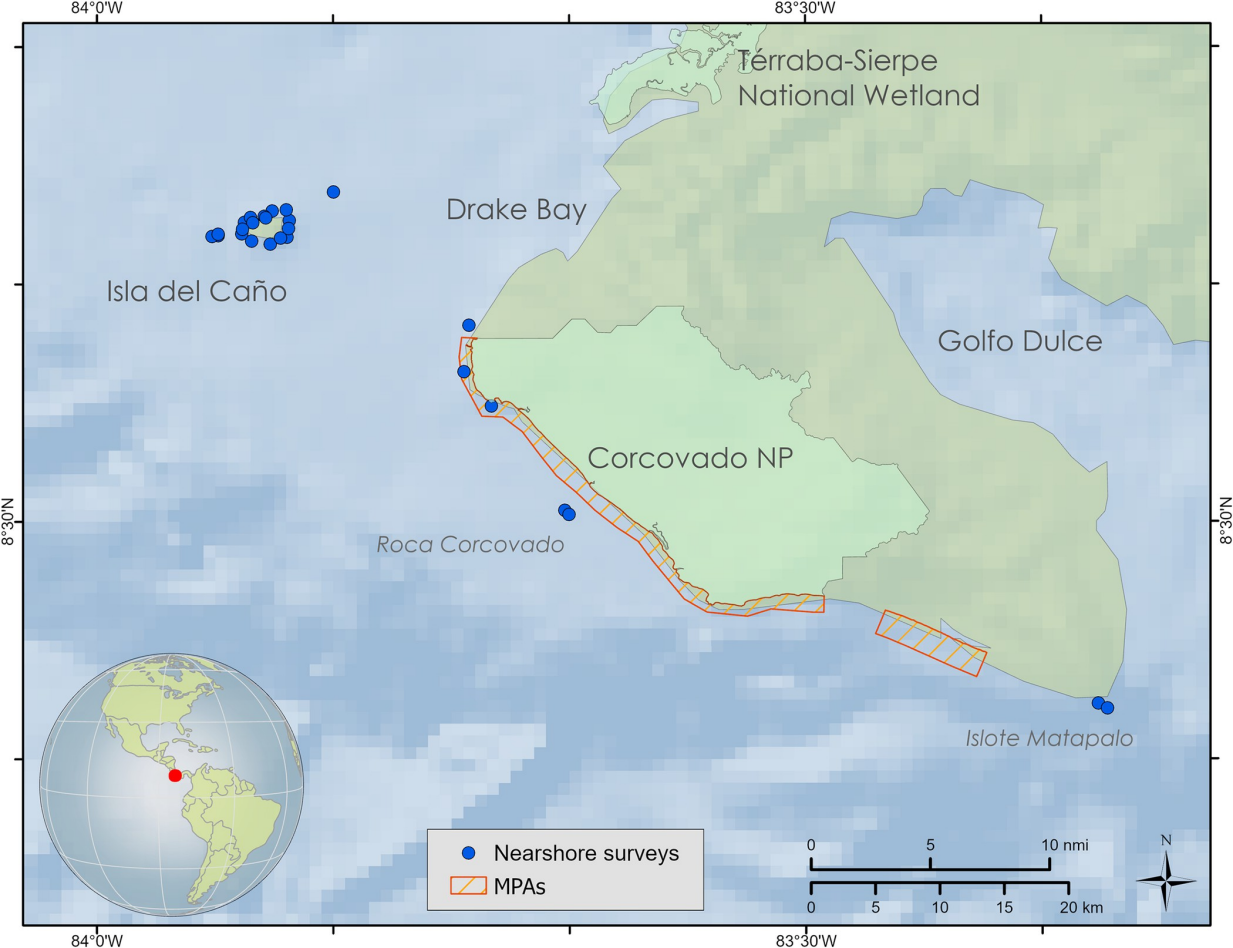
Locations visited on the expedition to the Osa Peninsula. Basemap derived from GEBCO Compilation Group (2020) GEBCO 2020 Grid (doi:10.5285/a29c5465-b138-234de053-6c86abc040b9). Processing and assembly of the Global Self-consistent, Hierarchical, High-resolution Geography Database for shoreline data from [32].

We conducted a total of 25 quantitative sampling surveys among three habitat types (IC, PIN, COAST) and at shallow (~ 10 m) and deep (~ 20 m) depth strata in most cases (N = 48), with 4 additional qualitative surveys along Osa Peninsula and Isla del Caño (Fig 2, S1 Table).

### Benthic communities

We identified a total of 95 different benthic taxa from 11 phyla, 17 classes, 35 orders, and 53 families around the Osa Peninsula (S2 Table). Turf algae accounted for 33.2% of total sessile benthic cover, followed by crustose coralline algae (CCA, 15.1%), the crustose calcareous alga *Peyssonnelia* sp. (7.1%), and the brown algae *Dictyota humifusa* (5.4%). Scleractinian hard corals accounted for 13% of total bottom cover, with *Porites lobata* (4.5%), *Pocillopora elegans* (3.9%), *Porites panamensis* (1.4%), and *Psammocora stellata* (1.2%) accounting for most of this assemblage. Soft corals (Alcyonacea) comprised 2.6% of total benthic cover with *Leptogorgia alba* and *Carijoa riisei* accounting for 78.7% of the total cover within this group. There was no significant difference in the number of benthic taxa among habitats (GLMM, χ^2^ = 3.483, p = 0.175), between depth strata (χ^2^ = 3.435, p = 0.064), or their interaction (χ^2^ = 3.273, p = 0.195).

Sessile benthic community structure based on cover by taxa was significantly different among major habitat types (Pseudo-F2,47−12.718, p = 0.001), with the differences between IC and PIN the most distinct (ANOSIM R = 0.856, p = 0.001), followed by PIN and COAST (ANOSIM R = 0.637, p = 0.001), with IC and COAST being most similar (ANOSIM R = 0.516, p = 0.001). Sessile benthic community structure was not significantly different between depth strata (Pseudo-F1,47−1.177, p = 0.300) or the interaction of depth and habitats (Pseudo-F2,47−1.021, p = 0.431). Sessile benthic cover by functional group also showed significant differences in community structure among habitats (Pseudo-F2,47−21.848, p = 0.001), but not between depths (Pseudo-F2,47−1.054, p = 0.358), or their interaction (Pseudo-F2,47−0.979, p = 0.423). Similar to the taxa level analysis, functional group assemblage structure was most distinct between IC and PIN (ANOSIM R = 0.860, p = 0.001), followed by COAST and PIN (ANOSIM R = 0.573, p = 0.001), and COAST and IC (ANOSIM R = 0.500, p = 0.001).

The average SIMPER dissimilarity of sessile benthic community structure between PIN and IC was 79.3%, with turf algae accounting for 21.6% of this dissimilarity and driven largely by the high cover of turf at IC ($\bar{X}$ = 38.7% ± 16.7) compared to the nearby PIN ($\bar{X}$ = 8.0% ± 17.5) (Table 1). The cover of CCA at PIN ($\bar{X}$ = 33.3 ± 16.7) was 3.5 times higher than at IC ($\bar{X}$ = 9.5 ± 8.1), contributing 16.0% to the total dissimilarity between these two habitats. *Peyssonnelia* sp. accounted for an additional 14.3% of the dissimilarity between these two habitats, with cover of this red calcareous alga at PIN ($\bar{X}$ = 24.9 ± 15.1) more than 10 times higher than at IC ($\bar{X}$ = 2.4 ± 3.1).

**Table 1:** Similarity of percentages (SIMPER) for benthic taxa most responsible for the percent dissimilarities among habitats using Bray-Curtis similarity analysis of hierarchical agglomerative group average clustering.

Avg. Diss. = 79.3	IC	PIN	Avg. Diss	% contrib.
Turf	38.7 (16.7)	8.0 (17.5)	17.2 (2.0)	21.6
CCA	9.5 (8.1)	33.3 (16.7)	12.7 (1.7)	16.0
*Peyssonnelia* sp.	2.4 (3.1)	24.9 (15.1)	11.3 (1.6)	14.3
*Lobophora* sp.	1.7 (4.9)	10.8 (14.0)	5.4 (0.9)	6.8
Avg. Diss. = 54.7	IC	COAST	Avg. Diss	% contrib.
Turf	38.7 (16.7)	39.8 (19.1)	10.0 (1.4)	18.2
CCA	9.5 (8.1)	14.6 (7.6)	4.9 (1.4)	8.9
*Caulerpa sertularioides*	7.7 (13.1)	-	3.8 (0.6)	7.0
*Porites lobata*	7.6 (7.6)	0.4 (0.7)	3.7 (1.0)	6.7
*Dictyota humifusa*	6.3 (6.1)	7.0 (5.3)	3.2 (1.4)	5.8
*Lobophora* sp.	1.7 (4.9)	6.0 (5.7)	3.1 (1.1)	5.6
*Pocillopora elegans*	6.2 (9.0)	0.3 (0.5)	3.1 (0.7)	5.6
Avg. Diss. = 68.0	PIN	COAST	Avg. Diss	% contrib.
Turf	8.0 (17.5)	39.8 (19.1)	17.8 (1.9)	26.2
CCA	33.3 (16.7)	14.6 (7.6)	10.7 (1.5)	15.7
*Peyssonnelia* sp.	24.9 (15.1)	4.5 (2.3)	10.3 (1.4)	15.1
*Lobophora* sp.	10.8 (14.0)	6.0 (5.7)	5.4 (1.0)	8.0

IC = Isla del Caño, PIN = pinnacles around Isla del Caño, and COAST = coastal rocky reefs and islets. Abundance values are average percent cover and one standard deviation in parentheses. Avg. Diss = Average dissimilarity with one standard deviation in parentheses. Only taxa with > 5% contribution to total dissimilarity are listed.

Benthic community structure between COAST and PIN also had high dissimilarity (68.0%), with turf algae nearly 5 times higher along the coast ($\bar{X}$ = 39.8% ± 19.1%) compared to PIN and accounted for 26.2% of the dissimilarity between these two habitats. CCA accounted for an additional 15.7% of the dissimilarity between these two habitats, with COAST cover ($\bar{X}$ = 14.5 ± 7.6) 2.3 times lower than at PIN. Dissimilarity between IC and COAST was 54.7%, with turf accounting for 18.2% of this difference although cover of this group was only 2.8% higher within COAST compared to IC. Two scleractinian hard corals (*Porites lobata* and *Pocillopora elegans*), accounted for 6.7% and 5.6%, respectively, of the dissimilarity between COAST and IC. Cover of *Porites lobata* was 21.0 times higher at IC ($\bar{X}$ = 7.6 ± 7.6) compared to COAST and *Pocillopora elegans* was 24.9 times higher at IC ($\bar{X}$ = 6.2 ± 9.0) compared to COAST.

**Fig 3:**
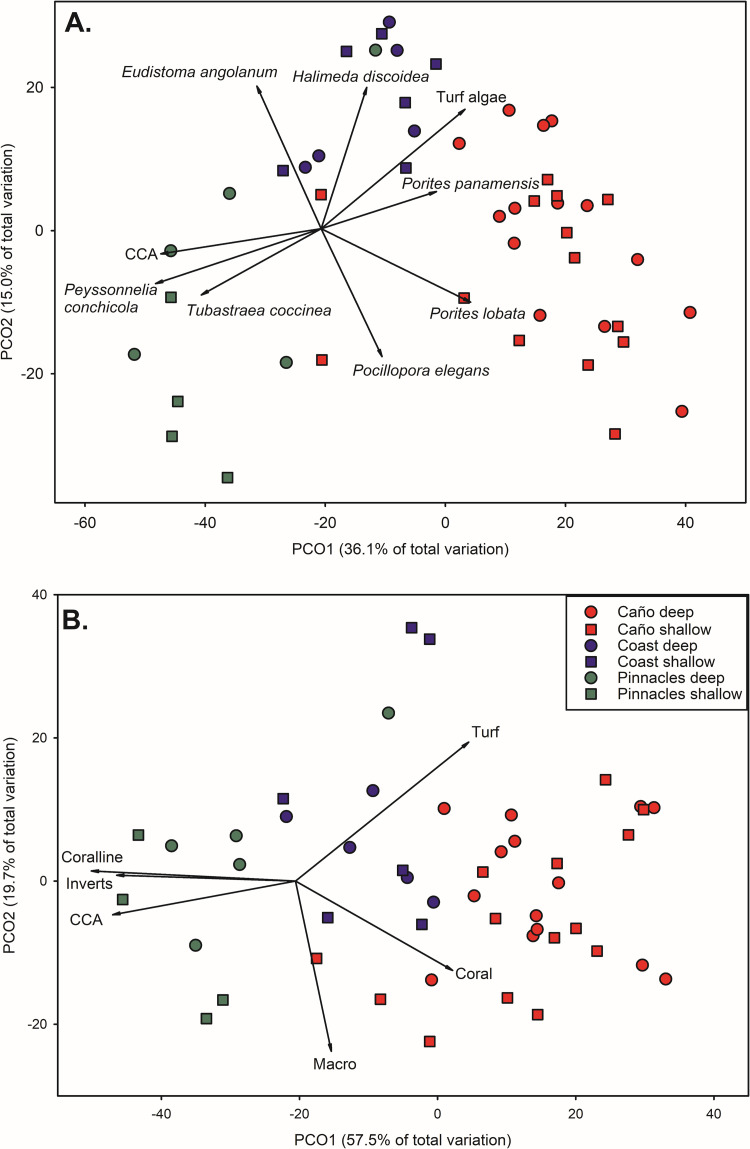
Principal coordinates analysis of benthic community composition based on percent cover. Data were arcsine-sqrt transformed prior to analyses. Vectors are the relative contribution and direction of influence of taxa to the observed variation among sites (Pearson product-moment correlations ≥ 0.5). A. Benthic community composition by taxa, B. Benthic community composition by functional group.

Sampling stations within habitat types based on benthic taxa were concordant with one another and separated in ordination space by habitat (Fig 3A). PCO1 accounted for 36.1% of total variation with locations around IC clustered towards the higher end of this axis and PIN clustering towards the lower end of the axis. *Peyssonnelia* sp. (*r* = -0.836), CCA (*r* = -0.809) and the orange cup coral *Tubastraea coccinea* (*r* = -0.604) were all highly correlated with PIN sites, while the scleractinian coral *Porites lobat*a (*r* = 0.728), turf algae (*r* = 0.713), and *P*. *panamensis* (*r* = 0.575) were highly correlated with IC sites. PCO2 explained an addition 15.7% of the variation in the benthic community. The calcareous green alga *Halimeda discoidea* (*r* = 0.595), the purple colonial tunicate *Eudistoma angolanum* (*r* = 0.583), and the sponge *Aplysina* cf. *revillagigedoi* (*r* = 0.575) were most closely correlated with COAST sites. *Pocillopora elegans* (*r* = -0.533) was correlated with sites around IC along PCO2 and *Balanus* sp. (*r* = -0.488) was most closely correlated with PIN stations along this same axis.

Sampling stations within habitat types based on benthic functional groups showed greater separated in ordination space by habitat compared to examination at the taxa level (Fig 3B). PCO1 accounted for 57.5% of the total variation and was positively correlated with turf algae (*r* = 0.739), and scleractinian coral (*r* = 0.674) towards IC. PCO1 was negatively correlated with crustose coralline algae (*r* = -0.901), CCA (*r* = -0.807), and other inverts (*r* = -0.785). An additional 19.7% of total variation was explained by PCO2 and negatively correlated with macroalgae (*r* = -0.709) towards shallow sites and positively correlated with turf algae (*r* = 0.588) towards deeper sites.

### Mobile invertebrates

There were 22 unique mobile invertebrate taxa recorded on transects from 3 phyla, 6 classes, 9 orders, and 15 families (S3 Table). Mollusca accounted for 59.1% of all taxa, followed by Echinodermata (36.4%), and Arthropoda (4.5%). The sea urchin *Hesperocidaris asteriscus* had the highest density among all mobile invertebrates ($\bar{X}$ = 73.3 ± 158.3 individuals 100 m^-2^) and occurred on 42% of transects. The long-spined sea urchin *Diadema mexicanum* was the most frequently encountered mobile invertebrate, occurring on 79% of transects with a density of 59.6 ± 58.6 indiv. 100 m^-2^. All other mobile invertebrates were in relatively low densities compared with these two sea urchin species.

Mobile invertebrate community structure was significantly different among major habitat types (Pseudo-F2,43−9.21, p = 0.001), between depths (Pseudo-F1,43−2.92, p = 0.022), and their interaction (Pseudo-F2,43−3.37, p = 0.002). The differences between IC and COAST were the most distinct (ANOSIM R = 0.530, p = 0.001), followed by IC and PIN (ANOSIM R = 0.315, p = 0.004), and PIN and COAST, which were indistinguishable from one another (ANOSIM R = 0.151, p = 0.051).

The average SIMPER dissimilarity of sessile benthic community structure between IC and COAST was 85.83%, with *D*. *mexicanum* accounting for 40.65% of this dissimilarity and *H*. *asteriscus* comprising an additional 31.58% (Table 2). Densities of *D*. *mexicanum* were 55.8% higher at IC compared with COAST while densities of *H*. *asteriscus* were 3.6 times higher at COAST sites. PIN and COAST showed 80.3% dissimilarity with *H*. *asteriscus* accounting for 44.91% of this dissimilarity, with densities at PIN sites 53.7% higher than COAST sites. IC and PIN were the least dissimilar (72.1%), but was also driven by *H*. *asteriscus*, which was 5.6 times more abundant at PIN sites compared to IC. *D*. *mexicanum* accounted for 37.7% of the dissimilarity between depths with densities in the shallow stratum 35.3% higher than deep stratum. *H*. *asteriscus* was slightly more abundant in the deep stratum ($\bar{X}$ 72.9 ± 204.4) compared to the shallow stratum ($\bar{X}$ 71.5 ± 123.1) and contributed an additional 33.2% to the dissimilarity between depth strata.

**Table 2:** Similarity of percentages (SIMPER) for mobile invertebrates most responsible for the percent dissimilarities among habitats using Bray-Curtis similarity analysis of hierarchical agglomerative group average clustering.

Avg. Diss. = 72.1	IC	PIN	Avg. Diss	% contrib.
*Hesperocidaris asteriscus*	29.7 (80.7)	166.0 (289.3)	30.8 (1.2)	42.7
*Diadema mexicanum*	68.6 (55.7)	52.0 (32.9)	16.5 (1.2)	22.9
*Hexaplex* sp.	3.4 (10.1)	16.0 (22.6)	6.6 (0.7)	9.1
Gastropoda 2	0.6 (3.0)	22.0 (37.2)	6.1 (0.6)	8.4
*Hexaplex princeps*	1.7 (9.1)	12.0 (23.8)	5.3 (0.6)	7.4
Avg. Diss. = 85.8	IC	COAST	Avg. Diss	% contrib.
*Diadema mexicanum*	68.6 (55.7)	44.0 (76.3)	34.9 (1.2)	40.7
*Hesperocidaris asteriscus*	29.7 (80.7)	108.0 (151.4)	27.1 (1.0)	31.6
*Pharia pyramidata*	0.6 (3.0)	10.7 (10.4)	7.0 (0.6)	8.1
*Hexaplex* sp.	3.4 (10.1)	4.0 (7.2)	4.5 (0.4)	5.2
Gastropoda 2	0.6 (3.0)	5.3 (7.9)	3.2 (0.5)	3.7
*Conus* sp.	0.0 (8.0)	4.0 (7.2)	1.6 (0.4)	1.9
Avg. Diss. = 80.3	PIN	COAST	Avg. Diss	% contrib.
*Hesperocidaris asteriscus*	166.0 (289.3)	108.0 (151.4)	36.1 (1.4)	44.9
*Diadema mexicanum*	52.0 (32.9)	44.0 (76.3)	17.0 (1.1)	21.2
Gastropoda 2	22.0 (37.2)	5.3 (7.9)	6.6 (0.6)	8.2
*Hexaplex* sp.	16.0 (22.6)	4.0 (7.2)	6.2 (0.7)	7.7
*Hexaplex princeps*	12.0 (23.8)	-	4.9 (0.5)	6.1
*Pharia pyramidata*	-	10.7 (10.4)	3.8 (0.7)	4.7

IC = Isla del Caño, PIN = pinnacles around Isla del Caño, and COAST = coastal rocky reefs and islets. Abundance values are means number of individuals per 100 m^2^ and one standard deviation in parentheses. Avg. Diss = Average dissimilarity with one standard deviation in parentheses.

**Fig 4:**
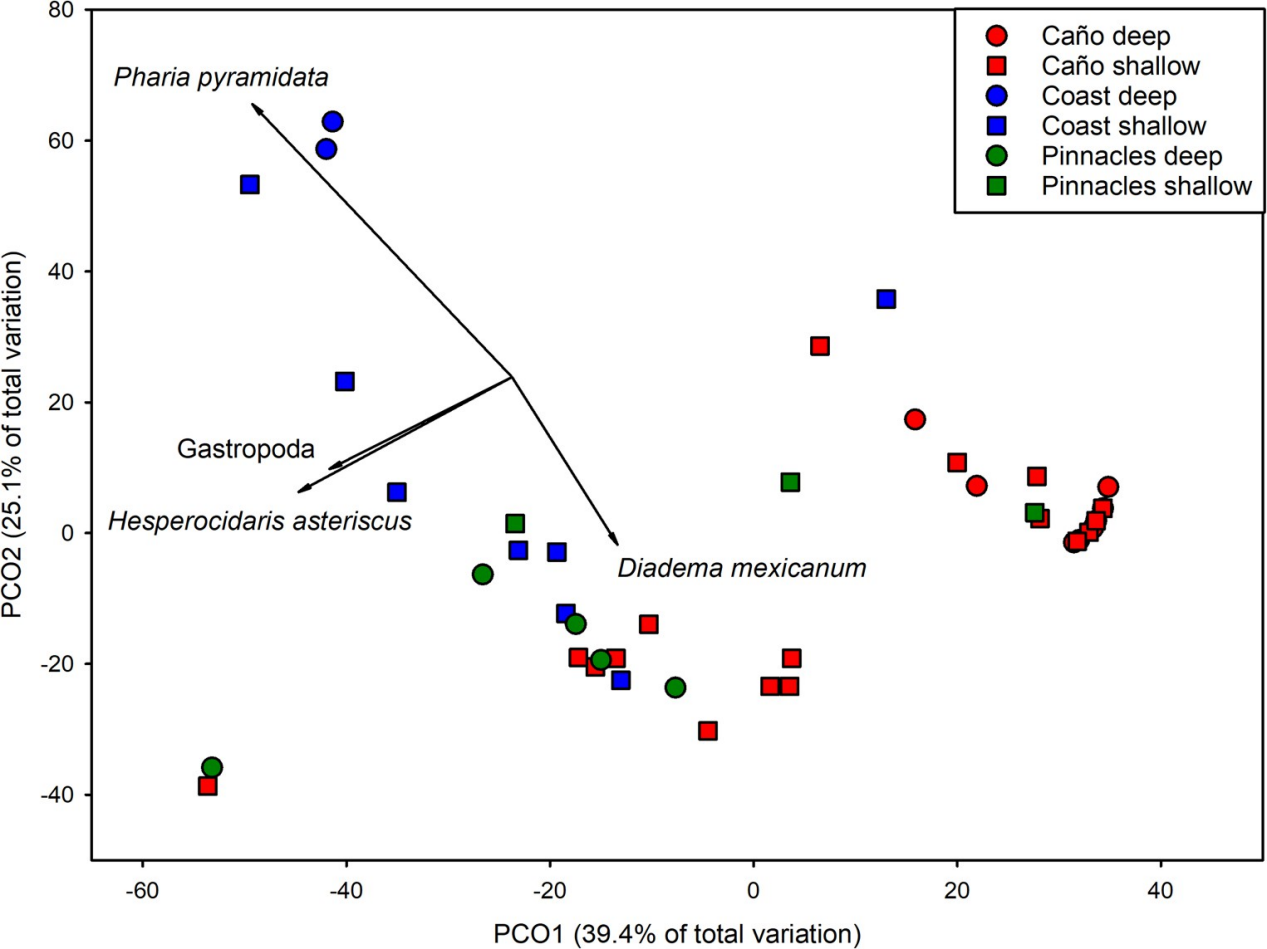
Principal coordinates analysis of mobile invertebrate assemblage structure composition based on numerical density (number of individuals 100 m^-2^). Data were ln(x+1)-transformed prior to analyses. Vectors are the relative contribution and direction of influence of taxa to the observed variation among sites (Pearson product-moment correlations ≥ 0.5).

Sampling stations within PIN where concordant with one another, while IC stations separated out by depth for the most part (Fig 4). COAST stations showed the most variability in ordination space. PCO1 accounted for 39.4% of total variation with PCO2 comprising an additional 25.1. *Pharia pyramidata* was negatively correlated with PCO1(*r* = -0.413) and positively corrected with PCO2 (*r* = 0.675). *H*. *asteriscus* was also negatively correlated with PCO1 (*r* = -0.340) and PCO2 (*r* = -0.300). *D*. *mexicanum* was strongly negatively correlated with PCO2 (*r* = -0.423). *H*. *asteriscus* was orthogonal to *P*. *pyramidata* and *D*. *mexicanum*, which were opposite one another in ordination space.

### Fishes

We observed a total of 129 different fish taxa during the expedition from 49 families (S4 Table). Of these, 112 taxa were recorded on quantitative transects. The most taxa rich families were Labridae (N = 10), Carangidae (N = 10), Lutjanidae (N = 8), Epinephelidae (N = 7), and Haemulidae (N = 7). Bluestriped chubs (*Kyphosus ocyurus*) were the most important species by weight, accounting for 16% of the total biomass but only occurring at 15% of the stations (S4 Table). Yellowfin surgeonfish, *Acanthurus xanthopterus*, was the next most important species by weight and was present at 58% of the stations. The razor surgeonfish (*Prionurus laticlavius*) and bigeye trevally (*Caranx sexfasciatus*) each comprised ~ 7% of total biomass. However, the razor surgeonfish was observed much more frequently (69% of the stations) compared with the bigeye trevally, which only was present at 23% of the stations.

**Fig 5:**
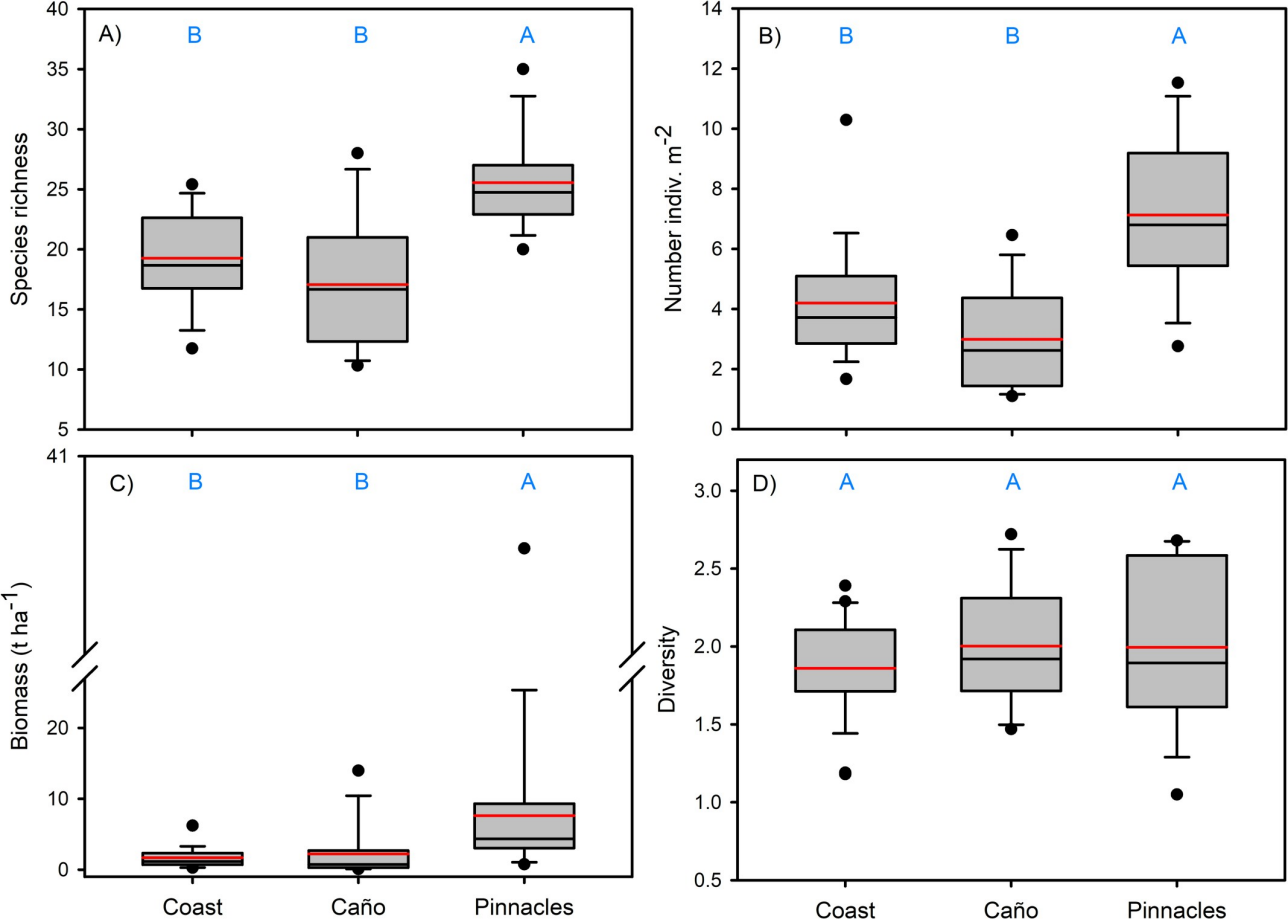
Comparisons of fish assemblage characteristics among habitats. Box plots showing median (black line), mean (red line), upper and lower quartiles, and 5th and 95th percentiles. A) Species richness transect, B) Number of individuals m^-2^, C) t ha^-1^, and D. Shannon-Wiener Diversity.

Overall mean fish taxa richness per transect was 20.4 (± 5.3) and was significantly higher at PIN compared to COAST and IC, which were not significantly different from one another (Table 3, Fig 5). Numerical abundance was significantly higher at PIN ($\bar{X}$ = 7.1 ± 2.5), compared to IC ($\bar{X}$ = 4.2 ± 2.2) and COAST (3.0 ± 1.6), which were not significantly different from one another. Fish biomass was significantly higher at PIN by 4.6 times ($\bar{X}$ = 7.9 ± 11.4) compared to IC ($\bar{X}$ = 1.7 ± 2.6) and 3.6 times higher than COAST ($\bar{X}$ = 2.2 ± 5.9).

**Table 3:** Comparisons of fish assemblage characteristics among habitats and depth strata.

Assemblage characteristic	Factor	Χ^2^	P	Contrasts
Species	Habitat	24.49	<0.001	Pinnacles > Caño = Coast
	Depth	1.11	0.203	
	Habitat x depth	3.12	0.210	
Number	Habitat	24.04	<0.001	Pinnacles > Caño = Coast
	Depth	0.29	0.587	
	Habitat x depth	0.84	0.657	
Biomass	Habitat	8.72	0.013	Pinnacles > Caño = Coast
	Depth	0.61	0.436	
	Habitat x depth	3.61	0.164	
Diversity	Habitat	0.43	0.803	Pinnacles = Caño = Coast
	Depth	0.01	0.999	
	Habitat x depth	2.49	0.289	

Results of Generalized Linear Mixed Models with Poisson distributions and log-link functions. Contrasts between fixed factors conducted using likelihood ratio tests.

Biomass between COAST and IC did not differ significantly from each other. Owing to its small size, only two stations were sampled at Islote Matapalo along the coast. However, this isolated rock had the highest biomass (8.9 ± 13.1 g m^-2^) observed during our surveys and was comprised of large schools of bluestriped chub (*K*. *ocyurus*), which accounted for 27% of the total biomass at this site. This was followed by the razor surgeonfish (*P*. *laticlavius*) and bigeye trevally (*C*. *sexfasciatus*), which accounted for an additional 22% and 18%, respectively, of the biomass at Islote Matapalo. Although fish biomass was more than 2 times higher in the deep depth stratum (4.5 ± 8.3) compared to the shallow depth stratum (2.2 ± 2.0) these differences were not significant. Diversity did not differ significantly among habitats, between depths, or their interaction. However, this metric showed the highest variability within PIN habitats and the least within COAST habitats.

The highest dissimilarity in fish assemblages based on taxa biomass was between COAST and PIN (SIMPER average dissimilarity = 92.0), with the greatest contribution to this dissimilarity coming from *C*. *sexfasciatus* (8.4%), followed by *Haemulon steindachneri* (8.0%), and *K*. *ocyurus* (7.9%) (Table 4). All these schooling species had much higher biomass at PIN stations compared to COAST. IC and COAST had an average dissimilarity of 88.1% and was driven by *Scarus rubroviolaceus* (9.6%), which had biomass 14.3 times higher at IC compared to COAST. Two species of schooling surgeonfishes, *P*. *laticlavius* and *A*. *xanthopterus*, accounted for an additional 7.3% and 7.0% of the dissimilarity between groups, respectively, with *A*. *xanthopterus* 15.2 times more abundant by weight at IC and *P*. *laticlavius* 3.5 times more abundant by weight at IC compared to COAST. Dissimilarity between PIN and IC was 85.7% and was driven by *H*. *steindachneri* (8.4%), which had a biomass of 70.5 g m^-2^ (± 176.1) at PIN but was not present on transects at IC. *Triaenodon obesus* was 3.7 times more abundant by weight at PIN vs. IC and contributed 7.9% to the dissimilarity between these groups. *Caranx sexfasciatus* accounted for an additional 6.6% of the dissimilarity between these two habitats and had a biomass of 63.4 g m^-2^ (± 155.5) at PIN stations but was not observed at IC. Similarly, biomass of *K*. *ocyurus* was three orders of magnitude greater at PIN 159.2 (± 551.4) vs. IC (0.8 ± 0.8) stations.

**Table 4:** Similarity of percentages (SIMPER) for fish biomass most responsible for the percent dissimilarities among habitats using Bray-Curtis similarity analysis of hierarchical agglomerative group average clustering.

IC & COAST Avg. Diss. = 88.1	IC	COAST	Avg. Diss. (SD)	Contrib %
*Scarus rubroviolaceus*	20.9 (20.4)	1.5 (5.3)	8.4 (1.0)	9.6
*Prionurus laticlavius*	9.9 (21.3)	34.7 (90.6)	6.5 (0.7)	7.3
*Acanthurus xanthopterus*	2.1 (4.2)	32.5 (71.2)	6.2 (0.5)	7.0
*Paranthias colonus*	10.8 (10.1)	6.4 (15.2)	4.7 (0.8)	5.3
*Caranx sexfasciatus*	0 (-)	33.1 (79.8)	4.2 (0.5)	4.8
IC & PIN Avg. Diss. = 85.68	IC	PIN	Avg. Diss. (SD)	Contrib %
*Haemulon steindachneri*	0 (-)	70.5 (176.1)	7.2 (0.4)	8.4
*Triaenodon obesus*	10.1 (29.4)	37.5 (49.2)	6.7 (0.8)	7.9
*Caranx sexfasciatus*	0 (-)	63.4 (155.5)	5.7 (0.4)	6.6
*Kyphosus ocyurus*	0.8 (0.8)	159.2 (551.4)	5.0 (0.4)	5.8
*Paranthias colonus*	10.8 (10.1)	37.6 (42.2)	4.6 (0.7)	5.4
COAST & PIN Avg. Diss. = 91.96	COAST	PIN	Avg. Diss. (SD)	Contrib %
*Caranx sexfasciatus*	33.1 (79.8)	63.4 (155.5)	7.8 (0.6)	8.4
*Haemulon steindachneri*	2.5 (9.2)	70.5 (176.1)	7.4 (0.4)	8.1
*Kyphosus ocyurus*	41.6 (111.8)	159.2 (551.4)	7.2 (0.5)	7.9
*Triaenodon obesus*	0 (-)	37.5 (49.2)	6.5 (0.7)	7.0
*Acanthurus xanthopterus*	32.5 (71.2)	66.7 (204.5)	5.8 (0.7)	6.3

IC = Isla del Caño, PIN = pinnacles around Isla del Caño, and COAST = coastal rocky reefs and islets. Biomass values are means g m^-2^ and one standard deviation in parentheses. Avg. Diss = Average dissimilarity with one standard deviation in parentheses.

**Fig 6:**
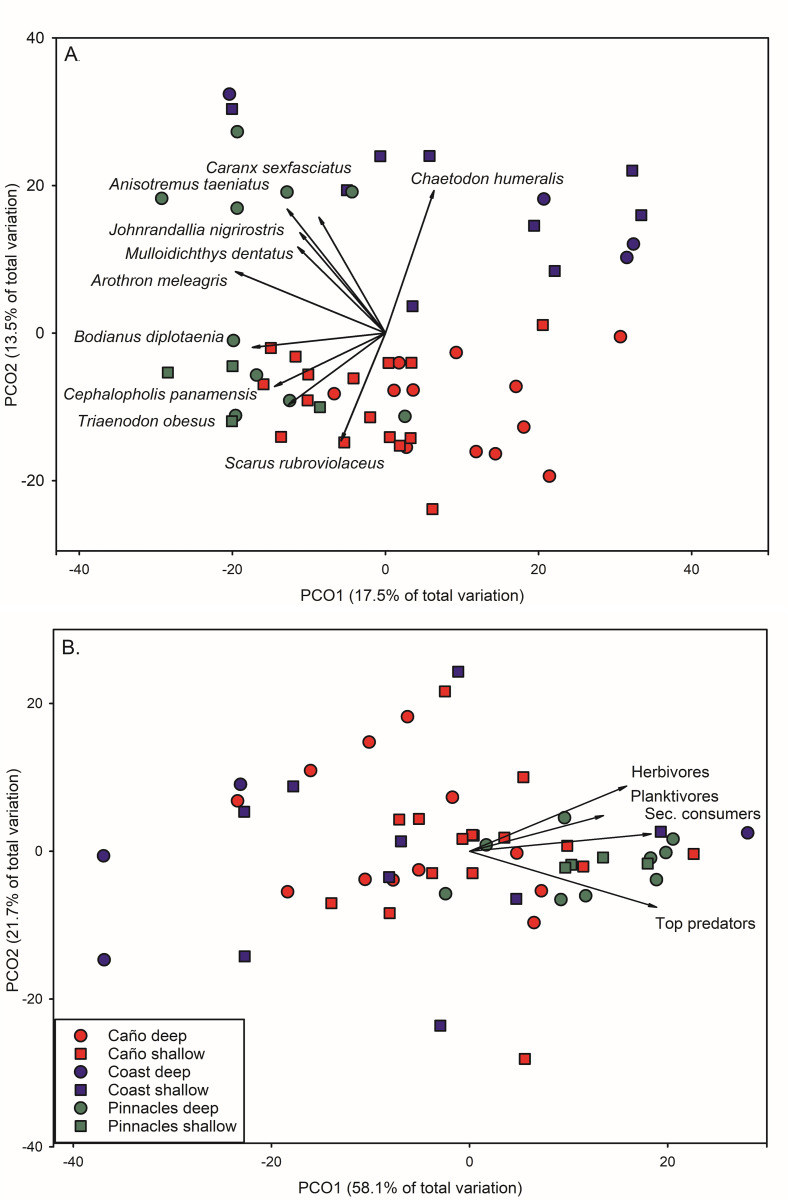
Principal coordinates analysis of fish assemblage structure based on biomass (g m^-2^). Data were 4^th^ root-transformed prior to analyses. Vectors are the relative contribution and direction of influence of taxa to the observed variation among sites (Pearson product-moment correlations ≥ 0.5). A. Fish assemblage structure based on taxa; B. Fish assemblage structure based on trophic group.

Fish taxa biomass assemblage structure was significantly different among habitats (pseudo-F_2,54_ = 5.64, p < 0.001) and between depths (pseudo-F_1,42_ = 2.14, p = 0.007), but not their interaction (pseudo-F_2,54_ = 1.26, p = 0.138). Fish assemblage structure between COAST and IC was most distinct (ANOSIM R = 0.613, p = 0.001), while IC and PIN were most similar (ANOSIM R = 0.381, p = 0.001). Fish assemblage structure based on taxa biomass showed separation in ordination space among habitats based on Principal Coordinates Analysis (Fig 6A). The 1^st^ PCO axis explained 17.9% of the variation in assemblage structure. This separation was driven by *Arothron meleagris* (*r* = -0.594) and *Bodianus diplotaenia* (*r* = -0.492), which were negatively correlated with PCO1 and trended towards PIN sites. PCO2 accounted for an additional 13.5% of the variation in fish assemblage structure with *Chaetodon humeralis* (*r* = 0.591), *Anisotremus taeniatus* (*r* = 0.577), and *Caranx sexfasciatus* (*r* = 0.470) the most highly correlated with this axis.

Fish trophic biomass assemblage structure was significantly different among habitats (pseudo-F_2,54_ = 7.42, p = 0.001), but not between depths (pseudo-F_1,54_ = 1.82, p = 0.141) or their interaction (pseudo-F_2,54_ = 0.82, p = 0.553). COAST vs. PIN trophic assemblages were overlapping but clearly different (R = 0.352), while COAST vs. IC and IC vs. PIN assemblages were barely separable (both R< 0.25). Trophic biomass assemblage structure showed separation in ordination space by habitats and depth based on Principal Coordinates Analysis (Fig 6B). The 1^st^ PCO axis explained 58.1% of the variation in assemblage structure and was positively correlated with all four trophic groups. Top predators had the strongest correlation with PCO1 (r = 0.561), followed by secondary consumers (*r* = 0.556), herbivores (*r* = 0.477), and planktivores (*r* = 0.400). PIN stations had high concordance and trends towards the higher end of PCO1. COAST stations trends towards the lower end of PCO1 but were more widely dispersed in ordination space.

## Discussion

The Osa Peninsula possesses the last large stand of tropical moist forest on the Pacific Coast of Mesoamerica and this nearly virgin rainforest extends all the way to the Pacific Ocean. Due to the high rainfall and steep terrain of Osa there are strong land-sea linkages that can have both positive (e.g., nutrient enrichment) and negative (e.g., sedimentation) effects on the marine environment. Much of the coastline around Osa is soft sediment and the extensive beach habitat provides critical nesting habitat for three species of threatened and endangered sea turtles–the Pacific green (*Chelonia mydas*), leatherback (*Dermochelys coriacea*), and olive ridley (*Lepidochelys olivacea*). The coastal rocky reefs of Osa have evolved in a high sediment and nutrient rich environment, which are dominated by turf, macroalgae, and crustose corallines, with low coral cover. The coastal islets and emergent rocks are exposed to high wave with benthic communities dominated by encrusting organisms and a relatively high cover of gorgonians, which are adapted to these harsh environmental conditions.

The lower reaches of the streams and rivers of Corcovado NP harbor a diverse assemblage of fishes, including a number of marine species [33], many of which were found on the coastal and offshore reefs during our study. These species feed on the abundant crustaceans, mollusks, and small fishes found in these habitats, which also serve as important nurseries for the juveniles of a number of these species. This strong connection between freshwater and marine environments of this region highlights the importance of healthy land/sea connections for ecosystem productivity in the area.

Approximately 15 km offshore from the peninsula is Isla del Caño, which has some of the most extensive coral reefs in the region [34] and the highest species richness of corals in Costa Rica [17, 21]. These reefs, like many in the region, suffered high mortality during the 1982–1983 El Niño event, but appear to have recovered to a large extent [21, 23, 35]. This unique ecosystem is a reservoir of biodiversity that may be resilient to near-term climate change.

The submerged pinnacles just outside the Isla del Caño Biological Reserve harbored unique invertebrate communities that includes the recent discovery of a new gorgonian [24], with high fish biomass, taxonomic richness, and abundance compared to nearby Isla del Caño and the coast. Fish biomass at these pinnacles was higher than most unprotected locations in the ETP [36] and comparable to iconic highly protected offshore MPAs in the region like Cocos Island National Park [37] and Malpelo [36, 38], as well as remote unfished reefs in the Pacific and Indian oceans [39]. Overall fish species richness from our quantitative surveys (N = 129) is higher than those reported from other parts of Costa Rica [40, 41] and similar to some well-studied areas in the region [42, 43].

Reefs in Golfo Dulce, a better studied nearby area, have been impacted by natural and anthropogenic disturbances. The decline of coral reefs in Golfo Dulce has resulted from increased sediments loads due to deforestation, slash-burn agriculture, road construction, and mining [3, 13]. There have also been increases in freshwater input, extreme ENSO events, and intense dinoflagellate blooms in the area [44].

The main source of sedimentation to the western portion of Osa and Isla del Caño is the Térraba River, which runs through the Térraba- Sierpe National Wetland—the largest mangrove estuary in Costa Rica [4, 16]. This ecosystem is an important nursery habitat for fishes and invertebrates that migrate to adjacent habitats along the coast as they mature [45, 46]. The considerable contribution of terrestrial sediments to the nearshore marine ecosystem has accelerated in recent years due to erosion of exposed lands, as well as coastal alteration [47]. These fine-grained sediments readily sticks to coral tissue and are more easily resuspended due to their lower density, contributing to reductions in water clarity for prolonged periods, locally reducing pH and oxygen conditions [48]. Coral cover along the coast of the Osa Peninsula is low and some locations appear to have suffered mortality from sedimentation [16]. Also, levels of bioerosion (destruction of the coral skeleton by organisms) are high in this region [13] and these coral reefs are being destroyed faster than they are accreting [49, 50].

Despite Isla del Caño Biological Reserve being fully protected from fishing by law, biomass was similar to fished areas along the coast and lower than the adjacent pinnacles, which are outside of the protected area. Illegal fishing and derelict fishing gear have been observed inside the reserve, particularly on the east side of the island, away from the ranger station. Owing to its high biodiversity and uniqueness, Isla del Caño experiences intensive ecotourism and these activities have the potential to negatively impact the reefs and associated fauna if not managed correctly.

Currently, the Corcovado National Park only include 500 m of ocean along the coast (20 km^2^ vs 422 km^2^ of protected land). Some of the main conservation interests of the national park are the high density of bull sharks (*Carcharhinus leucas*) in the estuarine environments and the cetaceans that transit the area [15]. Several rocks, islets, and islands (e.g., Islote Matapalo, Roca Corcovado) are currently not included within the park boundaries and these unique features represent important biodiversity hotspots, which should be included within the park and protected from fishing. There is limited enforcement of the marine portion of Corcovado NP, and we noted several instances of fishing within the park during our short expedition. Rough sea conditions limit fishing by boat to some extent so increased enforcement of the coastal zone could benefit local fish and invertebrate populations.

### Conservation

The unique configuration of healthy offshore coral reefs and pinnacles connected to coastal habitats around the Osa Peninsula provides corridors for many species including large predators such as sharks and other marine megafauna, which warrants additional protection. This is part of a larger corridor of MPAs that includes Isla del Coco National Park, the Galápagos Marine Reserve, Coiba National Park, and Malpelo Fauna and Flora Sanctuary. Oviedo and Solís [51] emphasize the need to ensure connectivity between key sites for marine mammals like humpback whales (*Megaptera novaengliae*), protecting a corridor between Isla del Caño and Drake Bay, as well as expanding the protected waters of Corcovado National Park. Also, the importance of both Golfo Dulce and Térraba-Sierpe National Wetland as reproductive sites for the scalloped hammerhead (*S*. *lewini*) have been demonstrated [12], and the southern Pacific coast of Costa Rica in general has been recognized for its important to endangered and threatened sea turtles [52].

The submerged pinnacles just outside the Isla del Caño Biological Reserve harbor extremely high fish biomass and unique biodiversity yet they are currently not protected within the Isla del Caño Biological Reserve, and fishing was evident as we found fishing lines on some of our dives. A logical conservation measure would be to expand the reserve to include these important ecological features. In addition, there is an urgent need for integrated ridge to reef management to reduce the impacts of coastal and road construction, discharge of effluent and pollutants into the coastal environment, illegal logging, and mining adjacent to Corcovado National Park.

Osa Peninsula is at a critical juncture. It is the last remaining section of Costa Rica’s Pacific coast where eco-tourism is the dominant type of tourism and a significant sector of the local economy [53]. Therefore, appropriate small-scale development must be balanced with sound environmental practices to provide sustainable social and economic opportunities. The area is relatively lightly populated, but previous land use and resource use practices have shifted these ecosystems into a compromised state, which from a water quality and marine resources perspective are far less productive than they might be under different future planning scenarios. A better understanding of ridge-to-reef connectivity is critically needed to ensure better management of nearshore ecosystem health [54–56]

Fisheries in the region are mainly artisanal, using hook-and-line, long-line, and trammel nets to target snappers, mackerel, and grunts [57]. However, there is a high bycatch of sharks, bony fishes, and turtles associated with the Costa Rican longline fishery and high catch rates of juvenile blacktip sharks (*Carcharhinus limbatus*) near the Osa Peninsula indicates that Osa is an important nursery area for this species [58]. Protection is urgently needed from this fishery in this area, which would also protect green and olive ridley turtles that nest on the Osa Peninsula. Shrimp trawling in Costa Rica has been shown to have significant environmental impacts on marine habitats and populations, including the bycatch of sub-reproductive sized fishes, as well as threatened and endangered species such as sea turtles [59, 60]. Shrimp trawling in Costa Rica is extremely unpopular with artisanal fishers and the creation of an MPA in the region that excluded this practice could benefit local fisheries [61]. Climate change and ENSO events have already impacted this region and while MPAs and integrated ridge to reef management cannot directly affect these processes, they can reduce local stressors and increase ecosystem resilience and resistance to climate change.

Considered one of the twenty-five most biodiverse countries of the planet, Costa Rica has earned global recognition for its extraordinary national parks and conservation of nature. Costa Rica is a founding member and co-chair of the High Ambition Coalition for Nature and People, whose objective is to set a global target to protect 30% of the planet’s sea and land by year 2030. Currently ~11% of the EEZ of Costa Rica is fully protected from fishing and other extractive activities, mostly within the Cocos Island National Park, which was recently expanded on December 17, 2021. The creation of a large MPA in the Osa region that includes the existing MPAs with greater protection and enforcement would benefit the rich biodiversity of this part of the country as well as replenishing nearby overexploited important fisheries resources.

## Supporting information

S1 Table Metadata for surveys conducted around Osa Peninsula, south Pacific Costa Rica. Geomor.–Geomorphic habitat types: sites around Isla del Caño (IC), 2) pinnacles around Isla del Caño (PIN), and 3) coastal rocky reefs and nearshore islets (COAST). Mangt.–Management type (no-take marine protected area, open to fishing). Stat.–stations (99# are qualitative surveys). CR–coral reef, P–pinnacle, IS–coastal islet, RR–rocky reef. No. benthic–number of benthic transects. No. fish–number of fish transects.
(DOCX)

S2 Table Benthic taxa identified during the expedition to Osa Peninsula. (DOCX)

S3 Table Macro invertebrate taxa observed on quantitative transects around Osa Peninsula. (DOCX)

S4 Table Fish species list from Osa Peninsula. Biomass based on quantitative surveys. See methods for a description. %Freq.–percent frequency of occurrence (n = 26 stations). Data type–Quan–quantitative, Qual–qualitative.
(DOCX)
