# Supplementary material for: Nearshore marine biodiversity of Osa Peninsula, Costa Rica: Where the ocean meets the rainforest
Source: PLoS One. 2022 Jul 28;17(7):e0271731. doi: 10.1371/journal.pone.0271731 (PMC9333237; doi:10.1371/journal.pone.0271731)
Supplement: S1 Table — Geomor.–Geomorphic habitat types: sites around Isla del Caño (IC), 2) pinnacles around Isla del Caño (PIN), and 3) coastal rocky reefs and nearshore islets (COAST). Mangt.–Management type (no-take marine protected area, open to fishing). Stat.–stations (99# are qualitative surveys). CR–coral reef, P–pinnacle, IS–coastal islet, RR–rocky reef. No. benthic–number of benthic transects. No. fish–number of fish transects. (DOCX) [file pone.0271731.s001.docx]

Table S1. Metadata for surveys conducted around Osa Peninsula, south Pacific Costa Rica. Geomor. – Geomorphic habitat types: sites around Isla del Caño (IC), 2) pinnacles around Isla del Caño (PIN), and 3) coastal rocky reefs and nearshore islets (COAST). Mangt. – Management type (no-take marine protected area, open to fishing). Stat. – stations (99# are qualitative surveys). CR – coral reef, P – pinnacle, IS – coastal islet, RR – rocky reef. No. benthic – number of benthic transects. No. fish – number of fish transects.

| Date | Location | Stat. | No. benthic | | No. fish | | Lat. | Long. | Habitat | Mangt. | Geomor. |
| --- | --- | --- | --- | --- | --- | --- | --- | --- | --- | --- | --- |
|  |  |  | 10m | 20m | 10m | 20m |  |  |  |  |  |
| 11-Mar-19 | Isla del Caño | 1 | 5 | 5 | 3 | 3 | 8.7103 | -83.8958 | CR | No-take | IC |
| 11-Mar-19 | Isla del Caño | 2 | 5 | 5 | 3 | 2 | 8.7183 | -83.8764 | CR | No-take | IC |
| 12-Mar-19 | Isla del Caño | 3 | 5 | 5 | 3 | 3 | 8.6971 | -83.8909 | CR | No-take | IC |
| 12-Mar-19 | Isla del Caño | 4 | 5 | 5 | 3 | 3 | 8.6951 | -83.8776 | CR | No-take | IC |
| 12-Mar-19 | Isla del Caño | 5 | 5 | 5 | 3 | 3 | 8.7116 | -83.8641 | CR | No-take | IC |
| 13-Mar-19 | Isla del Caño | 6 | 5 | 5 | 3 | 3 | 8.6998 | -83.8658 | CR | No-take | IC |
| 13-Mar-19 | Bajo del Diablo | 7 | 5 | 5 | 3 | 3 | 8.7010 | -83.9145 | P | No-take | PIN |
| 13-Mar-19 | Isla del Caño | 8 | 5 | 5 | 3 | 3 | 8.7144 | -83.8818 | CR | No-take | IC |
| 14-Mar-19 | Bajo Paraíso | 9 |  | 5 |  | 4 | 8.7315 | -83.8329 | P | Open | PIN |
| 14-Mar-19 | Isla del Caño | 10 | 5 | 5 | 3 | 3 | 8.7060 | -83.8647 | CR | No-take | IC |
| 14-Mar-19 | Isla del Caño | 11 | 5 | 5 | 3 | 3 | 8.7136 | -83.8915 | CR | No-take | IC |
| 15-Mar-19 | Cabo Matapalo | 12 | 5 | 5 | 1 |  | 8.3722 | -83.2937 | IS | Open | COAST |
| 15-Mar-19 | Islote Matapalo | 13 | 5 | 5 | 2 | 3 | 8.3687 | -83.2873 | IS | Open | COAST |
| 15-Mar-19 | Islote Matapalo | 999 |  |  |  |  | 8.3687 | -83.2873 | IS | Open | COAST |
| 16-Mar-19 | Roca Corcovado | 14 | 5 | 5 | 3 | 3 | 8.5079 | -83.6698 | IS | Open | COAST |
| 16-Mar-19 | Roca Corcovado | 15 | 5 | 5 | 3 | 3 | 8.5050 | -83.6670 | IS | Open | COAST |
| 17-Mar-19 | San Pedrillo | 16 | 5 | 5 | 6 |  | 8.6053 | -83.7411 | RR | No-take | COAST |
| 17-Mar-19 | San Josecito | 17 | 5 |  | 3 | 3 | 8.6380 | -83.7374 | RR | Open | COAST |
| 17-Mar-19 | Isla del Caño | 998 |  |  |  |  | 8.7100 | -83.8900 | CR | No-take | IC |
| 18-Mar-19 | Bajo Matador | 18 | 5 | 5 | 3 | 1 | 8.7003 | -83.9188 | P | No-take | PIN |
| 18-Mar-19 | Bajo del Diablo | 19 | 5 | 5 | 2 | 5 | 8.7020 | -83.9145 | P | No-take | PIN |
| 18-Mar-19 | Isla del Caño | 20 | 5 | 5 |  | 6 | 8.7022 | -83.8978 | CR | No-take | IC |

Table S1. Continued.

| Date | Location | Stat. | No. benthic | | No. fish | | Lat. | Long. | Habitat | Mangt. | Geomor. |
| --- | --- | --- | --- | --- | --- | --- | --- | --- | --- | --- | --- |
|  |  |  | 10m | 20m | 10m | 20m |  |  |  |  |  |
| 19-Mar-19 | Isla del Caño | 22 | 5 | 5 | 3 | 2 | 8.7053 | -83.8972 | CR | No-take | IC |
| 19-Mar-19 | Isla del Caño | 23 | 5 | 5 | 6 |  | 8.7133 | -83.8810 | CR | No-take | IC |
| 20-Mar-19 | Isla del Caño | 997 |  |  |  |  | 8.7020 | -83.9145 | CR | No-take | IC |
| 20-Mar-19 | Isla del Caño | 24 | 5 | 5 | 6 | 3 | 8.6993 | -83.8703 | CR | No-take | IC |
| 20-Mar-19 | Isla del Caño | 996 | 5 |  |  |  | 8.7020 | -83.9145 | CR | No-take | IC |
| 21-Mar-19 | Bajo del Diablo | 25 | 5 | 5 | 3 |  | 8.4691 | -83.7967 | P | No-take | PIN |
| 22-Mar-19 | Punta Llorona | 26 | 5 | 5 | 6 |  | 8.5814 | -83.7217 | RR | No-take | COAST |
