# Supplementary material for: Nearshore marine biodiversity of Osa Peninsula, Costa Rica: Where the ocean meets the rainforest
Source: PLoS One. 2022 Jul 28;17(7):e0271731. doi: 10.1371/journal.pone.0271731 (PMC9333237; doi:10.1371/journal.pone.0271731)
Supplement: S2 Table — (DOCX) [file pone.0271731.s002.docx]

Table S2. Benthic taxa identified during the expedition to Osa Peninsula.

| Phylum | Class | Order | Family | Taxa name |
| --- | --- | --- | --- | --- |
| Cyanobacteria | Cyanophyceae |  |  | Cyanobacteria unidentified |
| Cyanobacteria | Cyanophyceae | Oscillatoriales | Microcoleaceae | *Symploca* sp. |
| Chlorophyta | Ulvophyceae | Bryopsidales | Caulerpaceae | *Caulerpa cheminitzia* |
| Chlorophyta | Ulvophyceae | Bryopsidales | Caulerpaceae | *Caulerpa racemosa* |
| Chlorophyta | Ulvophyceae | Bryopsidales | Caulerpaceae | *Caulerpa sertularioides* |
| Chlorophyta | Ulvophyceae | Bryopsidales | Udoteaceae | *Chlorodesmis* cf. *hildebrandtii* |
| Chlorophyta | Ulvophyceae | Bryopsidales | Halimedaceae | *Halimeda* *discoidea* |
| Rhodophyta | Florideophyceae | Corallinales |  | Crustose coralline algae |
| Rhodophyta | Florideophyceae | Rhodymeniales | Champiaceae | *Champia* sp. |
| Rhodophyta | Florideophyceae | Corallinales | Lithophyllaceae | *Amphiroa* cf. *beauvoisi* |
| Rhodophyta | Florideophyceae | Corallinales | Lithophyllaceae | *Amphiroa* sp. |
| Rhodophyta | Florideophyceae | Gelidiales | Gelidiellaceae | *Gelidiella acerosa* |
| Rhodophyta | Florideophyceae | Gigartinales | Cystocloniaceae | *Hypnea pannosa* |
| Rhodophyta | Florideophyceae | Gigartinales | Cystocloniaceae | *Hypnea valentiae* |
| Rhodophyta | Florideophyceae | Nemaliales | Scinaiaceae | *Scinaia complanata* |
| Rhodophyta | Florideophyceae | Plocamiales | Plocamiaceae | *Plocamium* sp. |
| Rhodophyta | Florideophyceae | Rhodymeniales | Rhodymeniaceae | *Botryocladia* sp. |
| Rhodophyta | Florideophyceae | Rhodymeniales | Rhodymeniaceae | *Rhodymenia* sp. |
| Rhodophyta | Florideophyceae | Ceramiales | Rhodomelaceae | *Chondrophycus* sp. |
| Rhodophyta | Florideophyceae | Ceramiales | Rhodomelaceae | Rhodomelaceae Unidentified |
| Rhodophyta | Florideophyceae | Corallinales | Lithophyllaceae | *Amphiroa misakiensis* |
| Rhodophyta | Florideophyceae | Peyssonneliales | Peyssonneliaceae | *Peyssonnelia* sp. (calcified) |
| Ochrophyta | Phaeophyceae | Dictyotales | Dictyotaceae | *Dictyota* sp. 1 |
| Ochrophyta | Phaeophyceae | Dictyotales | Dictyotaceae | *Dictyota* sp. 2 |
| Ochrophyta | Phaeophyceae | Dictyotales | Dictyotaceae | *Dictyota* sp. 3 |
| Ochrophyta | Phaeophyceae | Dictyotales | Dictyotaceae | *Dictyota* *humifusa* |
| Ochrophyta | Phaeophyceae | Dictyotales | Dictyotaceae | *Lobophora* sp. |
| Ochrophyta | Phaeophyceae | Dictyotales | Dictyotaceae | *Padina* *durvillaei* |
| Ochrophyta | Phaeophyceae | Ectocarpales | Scytosiphonaceae | *Colpomenia* *sinuosa* |
| Ochrophyta | Phaeophyceae | Ectocarpales | Scytosiphonaceae | *Rosenvingea* *orientalis* |
| Porifera | Demospongiae |  |  | Encrusting beige sponge |
| Porifera | Demospongiae | Chondrosiida | Chondrosiidae | Chondrosia-like sponge |
| Porifera | Demospongiae | Clionaida | Clionaidae | *Cliona* sp. |
| Porifera | Demospongiae | Haplosclerida | Chalinidae | *Haliclona* sp. (orange) |

Table S2 continued.

| Phylum | Class | Order | Family | Taxa name |
| --- | --- | --- | --- | --- |
| Porifera | Demospongiae | Haplosclerida | Chalinidae | *Haloplegma duperreyi* |
| Porifera | Demospongiae | Poecilosclerida | Crambeidae | Crambe-like sponge |
| Porifera | Demospongiae | Poecilosclerida | Hymedesmiidae | *Phorbas* cf. *tenacior* |
| Porifera | Demospongiae | Verongiida | Aplysinidae | *Aplysina* cf. *revillagigedi* |
| Cnidaria | Anthozoa | Alcyonacea | Clavulariidae | *Carijoa riisei* |
| Cnidaria | Anthozoa | Alcyonacea | Gorgoniidae | *Leptogorgia alba* |
| Cnidaria | Anthozoa | Alcyonacea | Gorgoniidae | *Leptogorgia cofrini* |
| Cnidaria | Anthozoa | Alcyonacea | Gorgoniidae | *Leptogorgia rigida* |
| Cnidaria | Anthozoa | Alcyonacea | Gorgoniidae | *Pacifigorgia adamsii* |
| Cnidaria | Anthozoa | Alcyonacea | Gorgoniidae | *Pacifigorgia bayeri* |
| Cnidaria | Anthozoa | Alcyonacea | Gorgoniidae | *Pacifigorgia cairnsi* |
| Cnidaria | Anthozoa | Alcyonacea | Gorgoniidae | *Pacifigorgia firma* |
| Cnidaria | Anthozoa | Alcyonacea | Gorgoniidae | *Pacifigorgia irene* |
| Cnidaria | Anthozoa | Alcyonacea | Gorgoniidae | *Pacifigorgia rubicunda* |
| Cnidaria | Anthozoa | Alcyonacea | Plexauridae | *Heterogorgia verrucosa* |
| Cnidaria | Anthozoa | Alcyonacea | Plexauridae | *Muricea austera* |
| Cnidaria | Anthozoa | Scleractinia | Agariciidae | *Gardineroseris planulata* |
| Cnidaria | Anthozoa | Scleractinia | Agariciidae | *Pavona chiriquiensis* |
| Cnidaria | Anthozoa | Scleractinia | Agariciidae | *Pavona clavus* |
| Cnidaria | Anthozoa | Scleractinia | Agariciidae | *Pavona gigantea* |
| Cnidaria | Anthozoa | Scleractinia | Agariciidae | *Pavona varians* |
| Cnidaria | Anthozoa | Scleractinia | Dendrophylliidae | *Tubastraea coccinea* |
| Cnidaria | Anthozoa | Scleractinia | Pocilloporidae | *Pocillopora elegans* |
| Cnidaria | Anthozoa | Scleractinia | Poritidae | *Porites lobata* |
| Cnidaria | Anthozoa | Scleractinia | Poritidae | *Porites panamensis* |
| Cnidaria | Anthozoa | Scleractinia | Psammocoridae | *Psammocora stellata* |
| Cnidaria | Anthozoa | Zoantharia | Epizoanthidae | *Epizoanthus* sp. |
| Cnidaria | Hydrozoa |  |  | Hydraria unidentified |
| Cnidaria | Hydrozoa |  |  | Hydroidea unidentified |
| Cnidaria | Hydrozoa | Leptothecata | Aglaopheniidae | *Aglaophenia* sp. |
| Cnidaria | Hydrozoa | Leptothecata | Aglaopheniidae | *Macrorhynchia* *philippina* |
| Bryozoa | Gymnolaemata | Cheilostomatida | Bugulidae | *Bugula neritina* |

Table S2 continued.

| Phylum | Class | Order | Family | Taxa name |
| --- | --- | --- | --- | --- |
| Bryozoa |  |  |  | Encrusting white bryozoan |
| Mollusca | Bivalvia | Ostreida | Margaritidae | *Pinctada mazatlanica* |
| Mollusca | Bivalvia | Ostreida | Ostreidae | *Ostrea* sp. |
| Mollusca | Bivalvia | Ostreida | Ostreidae | *Striostrea* *prismatica* |
| Mollusca | Gastropoda | Littorinimorpha | Calyptraeidae | *Crepidula* sp. |
| Mollusca | Gastropoda | Littorinimorpha | Cypraeidae | Cypraeidae unidentified |
| Mollusca | Gastropoda | Littorinimorpha | Strombidae | *Aliger gigas* |
| Mollusca | Gastropoda | Littorinimorpha | Vermetidae | *Thylacodes* sp. |
| Mollusca | Gastropoda | Neogastropoda | Conidae | *Conus* sp. |
| Mollusca | Gastropoda | Neogastropoda | Fasciolariidae | *Fusinus* sp. |
| Mollusca | Gastropoda | Neogastropoda | Muricidae | *Hexaplex princeps* |
| Mollusca | Gastropoda | Neogastropoda | Muricidae | *Hexaplex* sp. |
| Mollusca | Gastropoda | Neogastropoda | Muricidae | Muricidae unidentified |
| Mollusca | Gastropoda | Neogastropoda | Turbinellidae | *Vasum* sp. |
| Mollusca | Gastropoda |  |  | Gastropoda unidentified |
| Mollusca | Gastropoda |  |  | Gastropoda unidentified |
| Arthropoda | Hexanauplia | Balanomorpha | Balanidae | *Balanus* sp. |
| Arthropoda | Malacostraca | Decapoda | Palinuridae | *Panulirus gracilis* |
| Echinodermata | Asteroidea | Valvatida | Acanthasteridae | *Acanthaster* *planci* |
| Echinodermata | Asteroidea | Valvatida | Mithrodiidae | *Mithrodia* *bradleyi* |
| Echinodermata | Asteroidea | Valvatida | Ophidiasteridae | *Pharia* *pyramidata* |
| Echinodermata | Asteroidea | Valvatida | Ophidiasteridae | *Phataria unifascialis* |
| Echinodermata | Echinoidea | Camarodonta | Toxopneustidae | *Tripneustes gratilla* |
| Echinodermata | Echinoidea | Cidaroida | Cidaridae | *Hesperocidaris asteriscus* |
| Echinodermata | Echinoidea | Diadematoida | Diadematidae | *Diadema mexicanum* |
| Echinodermata | Holothuroidea | Synallactida | Stichopodidae | *Isostichopus* *fuscus* |
| Chordata | Ascidiacea | Aplousobranchia | Didemnidae | Didemnidae unidentified |
| Chordata | Ascidiacea | Aplousobranchia | Didemnidae | *Diplosoma* *simile* |
| Chordata | Ascidiacea | Aplousobranchia | Polycitoridae | *Eudistoma angolanum* |
