# Supplementary material for: Nearshore marine biodiversity of Osa Peninsula, Costa Rica: Where the ocean meets the rainforest
Source: PLoS One. 2022 Jul 28;17(7):e0271731. doi: 10.1371/journal.pone.0271731 (PMC9333237; doi:10.1371/journal.pone.0271731)
Supplement: S3 Table — (DOCX) [file pone.0271731.s003.docx]

Table S3. Macro invertebrate taxa observed on quantitative transects around Osa Peninsula.

| Phylum | Class | Order | Family | Taxa |
| --- | --- | --- | --- | --- |
| Arthropoda | Malacostraca | Decapoda | Palinuridae | *Panulirus gracilis* |
| Echinodermata | Asteroidea | Valvatida | Acanthasteridae | *Acanthaster planci* |
| Echinodermata | Asteroidea | Valvatida | Ophidiasteridae | *Pharia pyramidata* |
| Echinodermata | Asteroidea | Valvatida | Ophidiasteridae | *Phataria unifascialis* |
| Echinodermata | Asteroidea |  |  | Asteroidea |
| Echinodermata | Echinoidea | Camarodonta | Toxopneustidae | *Tripneustes roseus* |
| Echinodermata | Echinoidea | Cidaroida | Cidaridae | *Hesperocidaris asteriscus* |
| Echinodermata | Echinoidea | Diadematoida | Diadematidae | *Diadema mexicanum* |
| Echinodermata | Holothuroidea | Synallactida | Stichopodidae | *Isostichopus fuscus* |
| Mollusca | Bivalvia | Ostreida | Margaritidae | *Pinctada mazatlanica* |
| Mollusca | Bivalvia | Ostreida | Ostreidae | *Ostrea* sp. |
| Mollusca | Bivalvia | Ostreida | Ostreidae | *Striostrea prismatica* |
| Mollusca | Gastropoda | Littorinimorpha | Cypraeidae | *Cypraea* sp. |
| Mollusca | Gastropoda | Littorinimorpha | Strombidae | *Aliger gigas* |
| Mollusca | Gastropoda | Neogastropoda | Conidae | *Conus* sp. |
| Mollusca | Gastropoda | Neogastropoda | Fasciolariidae | *Fusinus* sp. |
| Mollusca | Gastropoda | Neogastropoda | Muricidae | *Hexaplex princeps* |
| Mollusca | Gastropoda | Neogastropoda | Muricidae | *Hexaplex* sp. |
| Mollusca | Gastropoda | Neogastropoda | Muricidae | Muricidae unidentified |
| Mollusca | Gastropoda | Neogastropoda | Turbinellidae | *Vasum* sp. |
| Mollusca | Gastropoda |  |  | Gastropoda unidentified 1 |
| Mollusca | Gastropoda |  |  | Gastropoda unidentified 2 |
