# Supplementary material for: Nearshore marine biodiversity of Osa Peninsula, Costa Rica: Where the ocean meets the rainforest
Source: PLoS One. 2022 Jul 28;17(7):e0271731. doi: 10.1371/journal.pone.0271731 (PMC9333237; doi:10.1371/journal.pone.0271731)
Supplement: S4 Table — Biomass based on quantitative surveys. See methods for a description. %Freq.–percent frequency of occurrence (n = 26 stations). Data type–Quan–quantitative, Qual–qualitative. (DOCX) [file pone.0271731.s004.docx]

Table S4. Fish species list from Osa Peninsula. Biomass based on quantitative surveys. See methods for a description. %Freq. – percent frequency of occurrence (n= 26 stations). Data type – Quan – quantitative, Qual – qualitative.

| Family | Taxa | Biomass  (g m^-2^) (sd) | % Freq. | Data type |
| --- | --- | --- | --- | --- |
| Pomacentridae | *Abudefduf troschelii* | 2.08 (4.67) | 50 | Quan |
| Chaenopsidae | *Acanthemblemaria hancocki* | <0.01 | 76.92 | Quan |
| Chaenopsidae | *Acanthemblemaria macrospilus* | <0.01 | 3.85 | Quan |
| Chaenopsidae | *Acanthemblemaria exilispinus* | <0.01 | 53.85 | Quan |
| Acanthuridae | *Acanthurus nigricans* | 0.90 (1.45) | 69.23 | Quan |
| Acanthuridae | *Acanthurus triostegus* | 1.02 (2.94) | 19.23 | Quan |
| Acanthuridae | *Acanthurus xanthopterus* | 27.08 (82.66) | 57.69 | Quan |
| Carangidae | *Alectis ciliaris* | 0.53 (2.73) | 3.85 | Quan |
| Epinephelidae | *Alphestes immaculatus* | <0.01 | 3.85 | Quan |
| Epinephelidae | *Alphestes multiguttatus* | <0.01 | 7.69 | Quan |
| Monacanthidae | *Aluterus scriptus* | 0.02 (0.09) | 3.85 | Quan |
| Haemulidae | *Anisotremus interruptus* | 15.37 (75.33) | 11.54 | Quan |
| Haemulidae | *Anisotremus taeniatus* | 2.00 (4.55) | 50 | Quan |
| Apogonidae | *Apogon dovii* | 0.01 (0.03) | 19.23 | Quan |
| Apogonidae | *Apogon pacificus* | 0.06 (0.22) | 19.23 | Quan |
| Tetraodontidae | *Arothron hispidus* | 0.58 (1.25) | 50 | Quan |
| Tetraodontidae | *Arothron meleagris* | 0.98 (1.09) | 69.23 | Quan |
| Aulostomidae | *Aulostomus chinensis* | <0.01 | 3.85 | Quan |
| Tripterygiidae | *Axoclinus lucillae* | <0.01 | 42.31 | Quan |
| Pomacentridae | *Azurina atrilobata* | 3.43 (7.78) | 92.31 | Quan |
| Balistidae | *Balistes polylepis* | 1.14 (2.45) | 42.31 | Quan |
| Labridae | *Bodianus diplotaenia* | 2.95 (3.18) | 92.31 | Quan |
| Sparidae | *Calamus brachysomus* | 2.56 (13.08) | 3.85 | Quan |
| Balistidae | *Canthidermis maculata* | 0.28 (0.25) | 88.46 | Quan |
| Tetraodontidae | *Canthigaster punctatissima* |  |  | Qual |
| Carangidae | *Caranx caballus* | 0.70 (3.55) | 3.85 | Quan |
| Carangidae | *Caranx caninus* | 4.75 (24.24) | 3.85 | Quan |
| Carangidae | *Caranx lugubris* | 0.07 (0.36) | 3.85 | Quan |
| Carangidae | *Caranx melampygus* | 6.62 (11.93) | 61.54 | Quan |
| Carangidae | *Caranx sexfasciatus* | 20.69 (66.23) | 23.08 | Quan |
| Carcharhinidae | *Carcharhinus leucas* | 3.05 (15.57) | 3.85 | Quan |
| Epinephelidae | *Cephalopholis panamensis* | 1.06 (0.89) | 92.31 | Quan |
| Chaetodontidae | *Chaetodon humeralis* | 0.27 (0.42) | 57.69 | Quan |

Table S4 continued.

| Family | Taxa | Biomass  (g m^-2^) (sd) | % Freq. | Data type |
| --- | --- | --- | --- | --- |
| Chanidae | *Chanos chanos* |  |  | Qual |
| Cirrhitidae | *Cirrhitichthys oxycephalus* | <0.01 | 0 | Quan |
| Cirrhitidae | *Cirrhitus rivulatus* | 0.58 (1.05) | 57.69 | Quan |
| Gobiidae | *Coryphopterus urospilus* | <0.01 | 57.69 | Quan |
| Epinephelidae | *Dermatolepis dermatolepis* | 2.77 (14.13) | 3.85 | Quan |
| Diodontidae | *Diodon holocanthus* | 0.33 (0.95) | 15.38 | Quan |
| Diodontidae | *Diodon hystrix* | 0.46 (1.53) | 11.54 | Quan |
| Muraenidae | *Echidna nebulosa* |  |  | Qual |
| Chaenopsidae | *Ekemblemaria myersi* |  |  | Qual |
| Gobiidae | *Elacatinus puncticulatus* | <0.01 | 50 | Quan |
| Carangidae | *Elagatis bipinnulata* |  |  | Qual |
| Epinephelidae | *Epinephelus labriformis* | 3.9 (3.26) | 100 | Quan |
| Fistulariidae | *Fistularia commersonii* | 0.03 (0.1) | 19.23 | Quan |
| Gerreidae | *Gerres simillimus* | 0.6 (3.04) | 3.85 | Quan |
| Ginglymostomatidae | *Ginglymostoma unami* |  |  | Qual |
| Carangidae | *Gnathanodon speciosus* | 0.46 (1.62) | 7.69 | Quan |
| Muraenidae | *Gymnomuraena zebra* | 0.15 (0.76) | 3.85 | Quan |
| Muraenidae | *Gymnothorax castaneus* | 1.34 (2.71) | 30.77 | Quan |
| Haemulidae | *Haemulon flaviguttatum* | 0.43 (2.21) | 3.85 | Quan |
| Haemulidae | *Haemulon maculicauda* | 14.28 (39.11) | 34.62 | Quan |
| Haemulidae | *Haemulon melanurum* | 0.02 (0.07) | 26.92 | Quan |
| Haemulidae | *Haemulon sexfasciatum* | 6.9 (30.13) | 30.77 | Quan |
| Haemulidae | *Haemulon steindachneri* | 14.4 (44.17) | 15.38 | Quan |
| Labridae | *Halichoeres adustus* | <0.01 | 3.85 | Quan |
| Labridae | *Halichoeres chierchiae* | 0.26 (0.45) | 84.62 | Quan |
| Labridae | *Halichoeres dispilus* | 1.86 (1.8) | 100 | Quan |
| Labridae | *Halichoeres nicholsi* | 0.39 (0.5) | 80.77 | Quan |
| Labridae | *Halichoeres notospilus* | 0.17 (0.29) | 65.38 | Quan |
| Congridae | *Heteroconger klausewitzi* |  |  | Qual |
| Pomacanthidae | *Holacanthus passer* | 2.52 (3.82) | 88.46 | Quan |
| Lutjanidae | *Hoplopagrus guentherii* | 0.20 (1.03) | 3.85 | Quan |
| Dasyatidae | *Hypanus longus* | 1.55 (5.72) | 7.69 | Quan |
| Labridae | *Iniistius pavo* |  |  | Qual |
| Chaetodontidae | *Johnrandallia nigrirostris* | 0.79 (0.77) | 100 | Quan |
| Kyphosidae | *Kyphosus elegans* | 1.38 (1.97) | 61.54 | Quan |

Table S4 continued.

| Family | Taxa | Biomass  (g m^-2^) (sd) | % Freq. | Data type |
| --- | --- | --- | --- | --- |
| Kyphosidae | *Kyphosus ocyurus* | 54.56 (223.66) | 15.38 | Quan |
| Kyphosidae | *Kyphosus vaigiensis* | 2.57 (13.11) | 3.85 | Quan |
| Lobatidae | *Lobotes pacifica* |  |  | Qual |
| Lutjanidae | *Lutjanus argentiventris* | 11.85 (26.11) | 65.38 | Quan |
| Lutjanidae | *Lutjanus guttatus* | 3.26 (13.33) | 19.23 | Quan |
| Lutjanidae | *Lutjanus inermis* | 0.09 (0.28) | 11.54 | Quan |
| Lutjanidae | *Lutjanus jordani* | 0.18 (0.51) | 11.54 | Quan |
| Lutjanidae | *Lutjanus novemfasciatus* | 2.76 (6.36) | 19.23 | Quan |
| Lutjanidae | *Lutjanus peru* | 1.12 (5.7) | 3.85 | Quan |
| Lutjanidae | *Lutjanus viridis* | 1.16 (2.94) | 42.31 | Quan |
| Malacanthidae | *Malacanthus brevirostris* | 0.13 (0.61) | 11.54 | Quan |
| Labrisomidae | *Malacoctenus sudensis* | <0.01 | 15.38 | Quan |
| Chaenopsidae | *Mccoskerichthys sandae* | <0.01 | 30.77 | Quan |
| Balistidae | *Melichthys niger* | 0.11 (0.28) | 19.23 | Quan |
| Pomacentridae | *Microspathodon bairdii* | 0.10 (0.37) | 11.54 | Quan |
| Pomacentridae | *Microspathodon dorsalis* | 1.84 (2.56) | 57.69 | Quan |
| Mobulidae | *Mobula birostris* |  |  | Qual |
| Mobulidae | *Mobula thurstoni* |  |  | Qual |
| Mugilidae | *Mugil setosus* |  |  | Qual |
| Mullidae | *Mulloidichthys dentatus* | 1.98 (6.09) | 23.08 | Quan |
| Muraenidae | *Muraena lentiginosa* | 0.15 (0.64) | 15.38 | Quan |
| Epinephelidae | *Mycteroperca xenarcha* |  |  | Qual |
| Ophichthidae | *Myrichthys maculosus* | <0.01 | 0 | Quan |
| Ophichthidae | *Myrichthys xysturus* |  |  | Qual |
| Holocentridae | *Myripristis berndti* | 1.06 (2.55) | 30.77 | Quan |
| Holocentridae | *Myripristis leiognathus* | 1.24 (2.83) | 26.92 | Quan |
| Labridae | *Novaculichthys taeniourus* | 0.27 (0.41) | 50 | Quan |
| Blenniidae | *Ophioblennius steindachneri* | 0.13 (0.17) | 88.46 | Quan |
| Ostraciidae | *Ostracion meleagris* | 0.10 (0.25) | 23.08 | Quan |
| Cirrhitidae | *Oxycirrhites typus* | <0.01 | 3.85 | Quan |
| Epinephelidae | *Paranthias colonus* | 15.34 (17.31) | 96.15 | Quan |
| Blenniidae | *Plagiotremus azaleus* | 0.04 (0.03) | 96.15 | Quan |
| Pomacanthidae | *Pomacanthus zonipectus* | 0.12 (0.39) | 11.54 | Quan |
| Acanthuridae | *Prionurus laticlavius* | 22.34 (49.87) | 69.23 | Quan |
| Balistidae | *Pseudobalistes naufragium* | 3.24 (4.17) | 57.69 | Quan |

Table S4 continued.

| Family | Taxa | Biomass  (g m^-2^) (sd) | % Freq. | Data type |
| --- | --- | --- | --- | --- |
| Rhinobatidae | *Pseudobatos glaucostigma* | 0.25 (1.29) | 3.85 | Quan |
| Grammistidae | *Rypticus bicolor* | 0.02 (0.05) | 19.23 | Quan |
| Holocentridae | *Sargocentron suborbitale* | 0.07 (0.18) | 15.38 | Quan |
| Scaridae | *Scarus compressus* | 0.07 (0.33) | 3.85 | Quan |
| Scaridae | *Scarus ghobban* | 0.95 (1.98) | 38.46 | Quan |
| Scaridae | *Scarus perrico* | 0.74 (2.98) | 7.69 | Quan |
| Scaridae | *Scarus rubroviolaceus* | 13.18 (13.8) | 69.23 | Quan |
| Scorpaenidae | *Scorpaena histrio* | 0.02 (0.06) | 7.69 | Quan |
| Scorpaenidae | *Scorpaena mystes* | 0.01 (0.05) | 3.85 | Quan |
| Carangidae | *Seriola rivoliana* | 0.01 (0.06) | 3.85 | Quan |
| Serranidae | *Serranus psittacinus* | 0.12 (0.19) | 73.08 | Quan |
| Tetraodontidae | *Sphoeroides annulatus* | 0.01 (0.05) | 3.85 | Quan |
| Tetraodontidae | *Sphoeroides lobatus* | <0.01 | 3.85 | Quan |
| Sphyraenidae | *Sphyraena ensis* | 4.28 (21.81) | 3.85 | Quan |
| Sphyraenidae | *Sphyraena qenie* |  |  | Qual |
| Pomacentridae | *Stegastes acapulcoensis* | 0.11 (0.23) | 38.46 | Quan |
| Pomacentridae | *Stegastes flavilatus* | 1.23 (0.73) | 100 | Quan |
| Belonidae | *Strongylura* sp. |  |  | Qual |
| Balistidae | *Sufflamen verres* | 4.82 (3.13) | 100 | Quan |
| Synodontidae | *Synodus lacertinus* | <0.01 | 11.54 | Quan |
| Labridae | *Thalassoma grammaticum* | 0.03 (0.11) | 11.54 | Quan |
| Labridae | *Thalassoma lucasanum* | 5.68 (5.57) | 100 | Quan |
| Carangidae | *Trachinotus rhodopus* |  |  | Qual |
| Carcharhinidae | *Triaenodon obesus* | 14.07 (27.35) | 30.77 | Quan |
| Urotrygonidae | *Urobatis pardalis* | 0.12 (0.4) | 11.54 | Quan |
| Zanclidae | *Zanclus cornutus* | 0.73 (0.91) | 61.54 | Quan |
